# Exploring barriers to expanding medical training numbers in England: A national survey of medical education directors

**DOI:** 10.1016/j.clinme.2025.100523

**Published:** 2025-10-19

**Authors:** Michael Batavanis, Anmol Arora, Samuel Aryee, Nisha Nathwani

**Affiliations:** a School of Clinical Medicine, University of Cambridge, Cambridge, UK; b Addenbrooke’s Hospital, Cambridge, UK; c Department of Oncology, University College London, London, UK; d Bedfordshire Hospitals NHS Foundation Trust, Bedfordshire, UK; e Bedfordshire Luton and Milton Keynes ICB, NHS England Workforce, Training and Education East of England, Shefford, UK

**Keywords:** Resident doctors, Workforce expansion, Medical education, Clinical training, Specialty training

## Abstract

**Introduction:**

The UK faces a shortage of the medical workforce, which has prompted the NHS Long Term Workforce Plan to propose increases in medical training placements for resident doctors. Despite widespread agreement on the need for expansion, concerns persist regarding the ability of NHS trusts to provide adequate educator capacity, funding and infrastructure to support additional trainees. This study aims to identify the key barriers to trainee expansion as perceived by directors of medical education (DMEs) in England.

**Methods:**

Between June and August 2024, a cross-sectional survey was conducted of DMEs responsible for overseeing medical education within NHS trusts in England. Respondents were asked both quantitative and qualitative questions to elucidate the relative importance of challenges to trainee expansion, and the data were analysed using descriptive statistics and thematic analysis.

**Results:**

A total of 29 DMEs participated in the survey. The majority (93.1%) identified funding constraints as a critical barrier to trainee expansion, with many trusts struggling to meet financial demands despite NHS England salary contributions. Additionally, 89.7% of respondents reported inadequate educator capacity, and 72.4% cited a lack of suitable facilities to accommodate increased trainee numbers. Concerns were also raised regarding the ability to meet curricular and rotational requirements, particularly for higher specialty trainees.

**Discussion:**

There is an urgent need for trainee number expansion, but this study highlights challenges in expanding medical training placements, including non-financial barriers. Addressing educator shortages, ensuring sufficient funding and improving facilities are essential to successfully implementing workforce expansion plans.

## Highlights

• The study examines the barriers to expanding medical training placements in NHS trusts in England, as perceived by directors of medical education, in response to the NHS Long Term Workforce Plan’s proposed expansion of medical trainees.
• A cross-sectional survey conducted between June and August 2024 identified funding constraints as the most significant perceived barrier.
• Additional challenges include inadequate educator capacity and insufficient facilities, with concerns about meeting curricular and rotational requirements, particularly for higher specialty trainees.
• The findings also suggest disparities in training capacity across different NHS regions and between different specialties, with trusts struggling to provide essential resources such as office space, teaching facilities, and appropriate supervision for trainees.
• The study underscores the need for increased funding, enhanced educator support, and infrastructure improvements to ensure the successful implementation of workforce expansion plans and the sustainability of medical training in England.

## Graphical abstract

**Figure:**
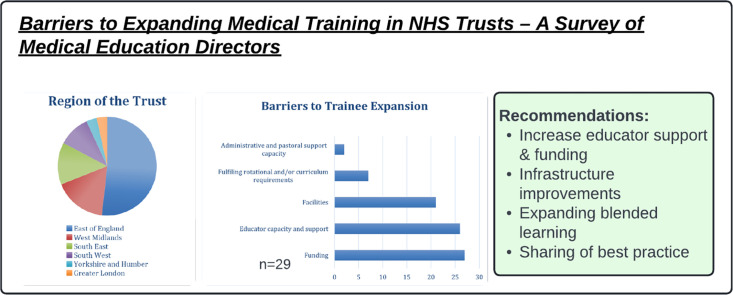

## Introduction

There are well-established concerns regarding the current shortage of doctors in the UK.^1^, ^2^, ^3^, ^4^ Recent statistics suggest that England has 3.2 doctors per 1,000 people, which is below the EU OECD (European Union Organisation for Economic Co-operation and Development) average of 3.9 doctors per 1,000 people.^3^ This is in the context of international healthcare workforce shortages, not limited to doctors.^5^ In recent years, there has been an influx of international medical graduates in England. The proportion of doctors in England with their primary medical qualification from a non-UK country has increased at more than three times the rate of those with a UK qualification.^3^ However, the UK government has sought to increase the number of doctors trained locally, with a plan to double the number of medical school places between 2023 and 2031.^6^ This has yet to be accompanied by a corresponding increase in training posts.

Strategies to improve the medical workforce crisis in England have been set out within the NHS Long Term Workforce Plan (2023), which outlines plans to address ‘*a shortfall of between 260,000 and 360,000 staff by 2036/37*’.^6^ The plan aims to increase the numbers of training doctors, alongside an increase in nurses and other healthcare professionals. Some of these goals include:

• doubling the number of medical school training places by 2031/32
• increasing the number of GP training places by 50% by 2031/32
• providing 22% of all training for clinical staff through apprenticeship routes by 2031/32, up from 7% in 2023. This includes introducing medical degree apprenticeships, with pilots running in 2024/25.

Despite general consensus that there is a need to expand the medical workforce, there has been some concern regarding the ability to develop educator capacity.^7^ This study presents a survey of medical educators in the UK to specifically answer the study question: ‘*What are the main barriers to trainee expansion in a trust?*’. By identifying concerns raised by directors of medical education (DMEs) regarding educator capacity, there can be a clear discussion to inform development and resources to enable implementation of trainee expansion across England in line with the NHS Workforce Long Term Plan.

## Methods

### Primary objective

To explore the barriers to meeting the needs of trainee expansion in NHS trusts across England.

### Survey design

The study involved a cross-sectional survey of educators conducted in June–August 2024, to understand issues relating to increasing capacity for doctors in training at all grades. The survey was disseminated to DMEs in England over an 8-week period in summer 2024.

### Survey participants

DMEs at trusts within England were approached to complete the survey. This cohort was selected as they are responsible within a trust for the quality of the learning environment provided by Local Education Providers (LEPs – mostly NHS trusts) for undergraduate and postgraduate medical education. They support the regional office and postgraduate dean in placements for doctors in training at all levels. They form an integral part of the team that develops medical workforce plans for their respective LEPs and have an insight into the crucial financial aspect, which may not be the case for training programme directors, as well as into discrepancies in facilities provided by individual trusts. The survey was emailed to all DMEs requesting completion via an online Google Form. DMEs were identified from internal directories and using publicly available foundation school websites.

### Survey development

This study builds upon previous research by conducting a national survey and maintaining a narrow focus on trainee expansion. Early research following the publication of the NHS Long Term Plan used a workshop approach to identify potential risks, including the expansion of medical school places, which was particularly topical at the time.^8^ This survey was developed by a working group within the NHS England (NHSE) Workforce, Training and Education team, involving trainee doctors, medical students, consultants and training programme directors. The ‘Medical Education Leaders UK’ consortium was also consulted for feedback on the survey design. The initial focus of the survey was educator capacity but, based on the feedback from the working group, it was agreed to broaden scope to focus on all barriers to expanding medical training numbers. Thus, the scope was expanded to identify which were the most important barriers overall, and to assess their relative importance. Due to the regional nature of training programmes, we also included questions where DMEs could comment on other trusts in their region, as they would either be aware of these or impacted by these. We also encouraged longer written answers to identify common themes and obtain suggestions for improvements, given their insight in the area.

The survey was hosted on Google Forms and accompanied by an email introducing the survey and team. Participants were asked to identify themselves in the survey. The survey instrument is included in Appendix 1.

### Data analysis

STROBE guidelines for cross-sectional studies were followed and the completed checklist is available in Appendix 2. Quantitative data analysis was conducted in Microsoft Excel using descriptive statistics, and the qualitative data were thematically analysed. Free-text responses from the survey were reviewed by members of the study team to identify common themes and recurring concerns raised by participants. This categorisation was done informally by consensus among the authors to generate illustrative themes and provide context for the quantitative findings. Where quotes are reported, these are attributed to job roles rather than individuals.

### Reflexivity

The authors of this study represent a diverse group of medical professionals whose roles included medical student, resident doctor and senior medical educator during the time of this study. Analysis was conducted by AA, MB and SA with supervision from NN. This mix of perspectives allowed incorporation of broad viewpoints.

## Results

**Fig. 1:**
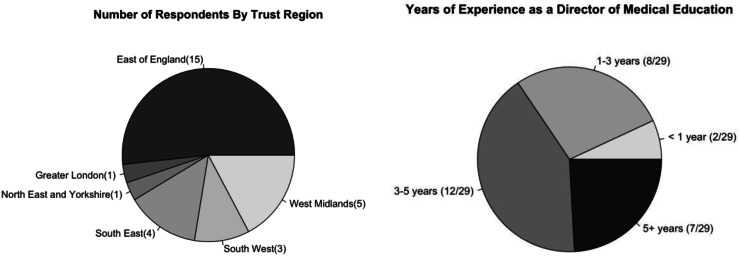
(Left) Region the DME’s trust is located in; (right) Number of years the respondent has worked in their current role as DME.

**Fig. 2:**
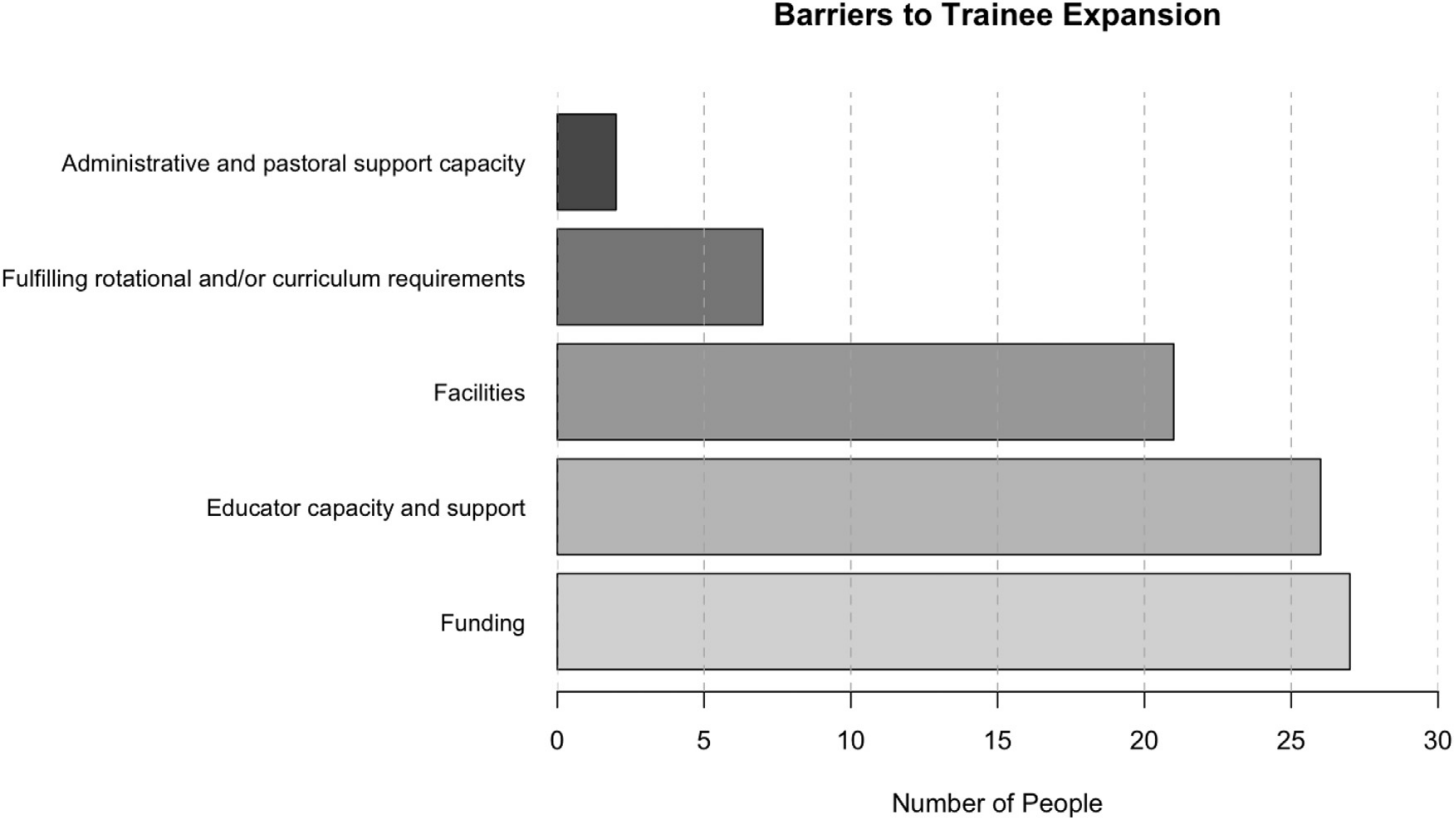
Perceived barriers to trainee expansion in each respondent’s respective trust.

Trusts in England are split into seven regions, all of which were represented apart from the North West. We received 29 responses covering trusts across England, but primarily originating from the East of England region (51.7%). The other major regions included were West Midlands (17.2%), South East (13.8%) and South West (10.3%). This survey represented 15 of the 22 trusts across the East of England (68%) and 29 of the approximately 207 NHS trusts (14%) in England. This includes hospital/acute trusts, mental health trusts, ambulance services trusts and community health trusts, although not all of these will host trainees. There was significant variation in the number of responses from each region and so comparisons are not made by region in this study. The majority of respondents (41.4%) had been in their role for 3–5 years, with 24.1% having been in this role for more than 5 years. Only 6.9% of respondents had been in this role for less than a year. Subgroup analysis by region is not presented due to small sample sizes, increasing the risk of identifying individual responses (Figs. 1 and 2).

Respondents were asked to select which factors would be the greatest challenge to expanding trainee numbers in their trust. A majority of DMEs (93.1%) believed funding to be one of the most critical issues. This was closely followed by lack of educator capacity and support (89.7%), and insufficient facilities (72.4%). Responders were also able to use free text to include any additional comments, which were collated into existing subgroups for analysis with additional subgroups added relating to rotational requirements (24.1%) and lack of administrative and pastoral support capacity (6.9%).

## Funding implications

Almost every respondent (96.6%) agreed that there would be funding implications with the proposed trainee expansion and explained their answer using free text. Many responses stated that, despite contribution to salary costs for expansion posts by NHSE, trusts were still not financially able to cover for the creation of new posts. In fact, five DMEs stated that their trusts have either put a freeze on new recruitment or will be cutting funding, despite being asked to take on more trainees. Four respondents also mentioned that medical workforce expansion is usually not accounted for in the budget setting of their trust. One respondent stated: ‘there still seems to be genuine surprise by managers that increasing doctor numbers might be needed. Educators and clinicians look forward to plan workforce but managers, in particular those in finance, look to the next quarter. This disconnect means that getting investment in medical workforce is a battle’.

Four respondents stated that trusts will need to convert existing locally employed doctor (LED) posts to training numbered posts, in order to achieve trainee expansion targets if they were required to by the regional dean. For trusts where there are currently no LEDs, any requirement for trainee expansion will require direct funding increases by the trust or support from the regional dean. One trust DME also raised concerns that, in an attempt to increase funding for expansion numbers, they would potentially have to lose more experienced LED posts to accommodate foundation doctor expansions. Foundation doctors are at the start of their training journey and need time to develop clinically; for departments where service delivery is essential, their ability to contribute is limited compared to an experienced LED. For foundation expansion, trusts need to identify 2 years’ funding so that they can support training for foundation year 1 and year 2.

There were suggestions made to try to overcome this barrier, which included having 100% of foundation year level basic salary being tariff-funded by NHSE or to encourage trusts to reduce locum costs. There have also been suggestions to support trusts to develop safer staffing models, which are derived centrally by modelling using population modelling and health inequalities.

### Educator capacity

From the survey, it was clear that another concern is around educator capacity. About 69% of respondents believed that, within their own trusts, there would not be adequate numbers of educators to support the trainee expansion outlined in the LTWP. Only 17.2% of respondents believed there was enough educator capacity to support expansion numbers (the remainder were unsure). This overlapped with responses in the next two sections, where both not enough educators and inadequate protected time to supervise doctors in training were reported.

### Facilities

DMEs were asked if there were specific facilities in their trust that would be affected by trainee expansion. 75.9% felt that their trust’s facilities would be inadequate to accommodate an expansion of trainee numbers, 13.8% felt that their facilities would be adequate and 10.3% were unsure. Respondents were prompted to explain their answers, and the common theme was that facilities are already lacking in many aspects without expansion, and with expansion this would get worse. When asked about other trusts, only 13.8% thought that other trusts in their region had adequate facilities.

Facilities regarded as being inadequate included office space, locker space, rest facilities, out-of-hours hot food, car parking, short-term accommodation, meeting rooms, simulation suites and IT infrastructure. 55.2% of respondents also raised concerns within clinical areas for doctors to have access to facilities including quiet rooms for patient discussion, computers and space to undertake patient-related admin and an environment that is supportive for staff to complete paperwork without disturbance or constant noise.

One DME raised the following concern: ‘The trust has asked us to stop taking all the F1s or all the F2s out for teaching on the same day as a group because of the impact it had on service provision, but we don’t have the training facilities or educator protected time to be able to do this’. They stated that they have raised this in their regional DME away day and that it seems to be a problem affecting all the trusts in their region.

### Rotational and/or curricular requirements

When asked if there are enough rotations to accommodate for the expanding number of trainees, 41.4% responded No, 37.9% responded Yes and 20.7% were unsure. Respondents were then asked if they thought other trusts in the same region would be unable to fulfil rotational and/or curricular requirements as a result of the workforce expansion: 27.6% agreed with this statement, 17.2% disagreed, and 55.2% were unsure. These rotational and/or curricular requirements appear to be a barrier to workforce expansion for higher specialty, as certain specific specialty requirements can only be met at tertiary units.

One respondent stated: ‘due to the facilities not keeping up pace with demand almost every trust in the region outsources to external companies – this is often the upper GI endoscopy work, which means it’s hard to provide training lists to trainees and clinical endoscopists without a facility and consultant workforce investment’. Consultant numbers to support and training of higher speciality trainees was considered inadequate. Although nurse trainers may be available to assist in teaching specific technical skills, this does not fulfil the wider brief of training or wider discussion of cases needed.

## Discussion

The expansion in trainee numbers has already begun, with a 25% increase in the number of medical school places taking place between 2018 and 2020. Five new medical schools have been approved from 2018 to 2020.^9^ Further increases are planned, with an additional 350 medical school places in England announced for the academic year 2025/26.^10^ The goal outlined in the NHS Long Term Workforce Plan is to further increase student numbers in the coming years – 10,000 by 2028/29 and a further 15,000 by 2031/32.^6^ This has led to the UK Foundation Programme being oversubscribed in 2022, with 791 students being placed on a reserve list, an increase from 25 in 2017.^11^ This is also heavily impacted by increasing numbers of international applicants to the programme. The foundation schools are working on solutions for the increased numbers of foundation doctors, with excess applicants being provided with ‘placeholder jobs’ from 2023, with around 10% of the 9,702 applicants in 2024 estimated to have received these.^12^ To meet the requirement for more higher training posts once these increased foundation doctors complete foundation training, there is a parallel increase in specialty training posts, with an increased number of 11,207 doctors accepting training programme posts in 2022/23 and 876 new posts being announced by NHSE in 2023.^13^ Conversations are ongoing about the urgent necessity to increase numbers of specialty training posts. The NHS Workforce Plan recognised the need to increase GP training numbers by approximately 50% within the next decade, but other specialties are awaiting firm commitments. Competition ratios are increasing rapidly, with 2024 figures revealing 5.35:1 for ST1 entry training posts and 3.95:1 for ST3 training posts. Application numbers for these entry levels increased by 48.6% and 14.9% respectively within a single year.^14^^,^^15^ It should be noted that these numbers are likely an overestimate in competition, considering that some applicants will apply for more than one post and later recruitment rounds within the same application cycle will therefore have lower competition ratios.

There continues to be concern regarding the ability of trusts across England to cope with increasing expansion numbers. As outlined, this relates primarily to funding issues, educator capacity problems, facilities and training opportunities, especially for higher specialty trainees. This survey, despite its limitations, suggests that funding was the main concern of respondents as a key barrier to trainee expansion. NHSE provides substantial support for salary costs of resident doctors, including covering 100% of foundation trainee base salary when educational grants are accounted for. In addition to salary contributions, NHSE pays for education support costs, study leave, relocation and excess travel for all specialty training posts in England.^16^ However, naturally there will be supplementary costs that trusts must meet as their workforce expands, even if salaries are reimbursed via central funding. Ongoing strain on NHS budgets may be impacting on their ability to support the expansion of posts. Many trusts around the country are struggling to reach their efficiency targets and are planning to freeze or cut posts to save money.^17^

The NHS Long Term Workforce Plan itself also sets out plans to increase funding to enable trainee expansion, with more than £2.4 billion of additional funds stated to be cumulatively invested over the 2023/29 period, to support a 27% expansion in training places.^6^ Another initiative, the Addressing Health Inequalities: Distribution of Medical Specialty Training Programme workstream aims to ensure equitable distribution of NHSE-funded specialty training posts across England to help tackle health inequalities.^18^ They acknowledge the mismatch between the current distribution of posts and the actual demand of health services delivered by doctors in training is not aligned to balance the health inequalities across England.^19^

In order for the NHS to be able to cope with the increasing demands on it, we need to retain and maximise the productivity of our current workforce, and sustainably train a high-quality workforce for the future. To achieve this, there needs to be adequate support for education and training, and NHSE published its Educator Workforce Strategy in March 2023 to outline an approach towards this.^20^ As detailed in the report, this is meant to complement the Long Term Workforce Plan, as it is expected that the demand on trainers will only continue to grow. They acknowledge that one of the key issues is that ‘service pressures have eroded the time available for both supervising learners and supporting their wellbeing’.

According to the General Medical Council (GMC) National Training Survey 2024, which surveyed over 18,000 educators, the large majority (90%) of trainers enjoy their role.^21^ However, over a quarter (27%) do not think that they have enough time allocated for their role as an educator as part of their job plan. The respondents in this survey (89.7%) agreed with these concerns of insufficient educator capacity and support for the planned workforce expansion. One respondent to our survey mentioned that we need to recruit more senior doctors to support the expanding cohort of incoming junior doctors. This highlights the need to address issues such as the burnout faced by educators, trust funding concerns and appropriate time and support for the consultants who are able to be educational supervisors.

Insufficient facilities were the third most selected barrier to trainee expansion in our survey (72.4%). Many respondents stated that facilities in their trusts were already lacking, and the specific examples given varied from trust to trust. In some cases, respondents mention departments where there is physically not enough space to accommodate new doctors. There are concerns about educational facilities, including the need for simulation faculty and technology-enhanced learning. The variability in facilities across England is linked to trust funding and how medical education funding is supported in individual trusts.

The main limitation of our study was the small sample size of 29, and the variable representation between regions across England. The majority of responses were from the East of England, where the study team are based, which limits comparison between different regions across England. The study was cross-sectional in nature, so we are unable to comment on dynamic changes within trusts or regions.

## Concluding remarks

This limited survey of senior medical educators (DMEs) reviews the factors that impact the placement of training doctor expansion in England. The study has identified a range of barriers to expansion of the number of doctors in England, with funding and educator faculty capacity cited as the most commonly perceived barriers to expansion. Practical recommendations include investment in educational infrastructure, such as simulation and technology-enhanced learning faculties. Availability of supervisors was also flagged as a key issue, with potential solutions including an increase in the number of hours contracted for educational supervision, although the definitive solution might be a concurrent increase in consultant posts. Naturally, the most commonly raised barrier to trainee expansion was funding and future studies may seek to focus on the economic analysis of increasing trainee numbers, in the short and long term. As well as the potential solutions emerging from this study, ideas for future research may include expanding blended learning and sharing of best practice between organisations. There is also a shortage of research focusing on the post-CCT careers of trainees and the characterisation of the adequacy of consultant and GP posts.

## Ethics approval and consent to participate

Ethical approval was not required for this cross-sectional survey of NHS staff. Patients, or patient data, were not directly involved in the study. According to UK Health Research Authority guidance, ‘research involving NHS or social care staff recruited as research participants by virtue of their professional role’ does not normally require review by an NHS Research Ethics Committee. Participation was voluntary, informed consent was implied by survey completion, and responses were anonymised during analysis. Participants were informed about the context of the study and that anonymised responses would be published and presented in an aggregate format.

## Funding

This research did not receive any specific grant from funding agencies in the public, commercial or not-for-profit sectors.

## Data availability statement

The qualitative data that support the findings of this study are available from the corresponding author upon reasonable request.

## CRediT authorship contribution statement

**Michael Batavanis:** Writing – original draft, Methodology, Investigation, Conceptualization. **Anmol Arora:** Writing – original draft, Methodology, Investigation, Conceptualization. **Samuel Aryee:** Writing – review & editing, Methodology, Investigation, Conceptualization. **Nisha Nathwani:** Writing – review & editing, Supervision, Project administration, Methodology, Investigation, Conceptualization.

## Declaration of competing interest

The authors declare that they have no known competing financial interests or personal relationships that could have appeared to influence the work reported in this paper.

## Appendix Supplementary materials

Image, application 1

## References

[1] Brennan N., Langdon N., Gale T., Humphries N., Knapton A., Bryce M. (2023). Exploring recent patterns of migration of doctors to the United Kingdom: a mixed-methods study. BMC Health Serv Res.

[2] Platts D. (2023). The NHS doctors staffing crisis in numbers. BDIRESourcing.

[3] British Medical Association (2025).

[4] Taylor M. (2020). Why is there a shortage of doctors in the UK?. Bullet Royal Coll Surg Engl.

[5] Boniol M., Kunjumen T., Nair T.S., Siyam A., Campbell J., Diallo K. (2022). The global health workforce stock and distribution in 2020 and 2030: a threat to equity and ‘universal’ health coverage?. BMJ Global Health.

[6] NHS England (2023). NHS long term workforce plan. NHS Engl.

[7] Shembavnekar N., Kelly E., Charlesworth A. (2023). How feasible are the NHS Long Term Workforce Plan commitments on training?. The Health Found.

[8] Geary U., McKee M., Petty-Saphon K. (2024). Mind the implementation gap: a systems analysis of the NHS Long Term Workforce Plan to increase the number of doctors trained in the UK raises many questions. Br Med Bull.

[9] Department of Health and Social Care and The Rt Hon Steve Barclay MP (2023).

[10] Department of Health and Social Care, NHS England, The Rt Hon Victoria Atkins MP and The Rt Hon Gillian Keegan (2024).

[11] Lok P. (2022). UK’s foundation training programme for 2022 was oversubscribed by almost 800 places. BMJ.

[12] Tucker R. (2024). Rapid response: placeholder jobs not only isolate doctors, they are detrimental to the retention crisis [online]. BMJ.

[13] NHS England Workforce, Training and Education (2023).

[14] Specialty Applications (2025). ST3 Specialty training competition ratios. Spec Applic.

[15] Specialty Applications (2025). CT1 & ST1 Specialty training competition ratios. Spec Applic.

[16] NHS England Workforce, Training and Education (2024).

[17] Barron J. (2024).

[18] NHS England Workforce, Training and Education (2024).

[19] NHS England Workforce, Training and Education (2024).

[20] NHS England (2023).

[21] General Medical Council (2024).

