# Supplementary material for: Exploring barriers to expanding medical training numbers in England: A national survey of medical education directors
Source: Clin Med (Lond). 2025 Oct 19;25(6):100523. doi: 10.1016/j.clinme.2025.100523 (PMC12639427; doi:10.1016/j.clinme.2025.100523)
Supplement: Supplementary file 1 [file mmc1.docx]

**Appendix 1: Survey Instrument**

**Survey Instrument**

The questions asked in the survey to Directors of Medical Education were as follows:

1. *Name*
2. *Email*
3. *In which region is your trust based?*
4. *Name of your trust?*
5. *For how long have you been in your role as a Director of Medical Education?*
6. *Do you believe there is enough educator capacity within your trust to accommodate for trainee expansion, as will be expected under the Long-Term Workforce Plan (LTWP)?*
7. *With regards to trainee expansion within your trust, what do you feel would be the main difficulties?*
   1. *Options include:*
      1. *Educator Capacity & Support*
      2. *Fulfilling curriculum requirements*
      3. *Funding*
      4. *Facilities*
      5. *Rotational requirements*
      6. *Other*
      7. *If you selected other, please provide more details:*
8. *Are there likely to be funding implications due to the trainee expansion?*
   1. *If yes, more details:*
9. *Are you aware of any trusts within your region lacking adequate facilities for trainees? (i.e. office space, rest facilities, access to food, access to lockers etc.)*
10. *Are there any facilities specific to your trust that may be affected by the trainee expansion?*
    1. *If yes, more details:*
11. *Are there enough rotations to accommodate the new number of trainees?*
12. *Are there any specific trusts you envision may be unable to fulfill rotational and/or curricular requirements as a result of the workforce expansion?*
    1. *If yes, more details:*
13. *Do you envision that provision for locally employed doctors (LEDs) will be affected by trainee expansion?*
14. *Do you envision that provision for medical students will be affected by trainee expansion?*
15. *Have you raised any concerns regarding trainee expansion?*
16. *If you had concerns, who would you report them to?*

**Appendix 2: STROBE Checklist for Cross-Sectional Studies**

**​​STROBE Statement—Checklist of items that should be included in reports of *cross-sectional studies***

|  | Item No. | Recommendation | Page No. |
| --- | --- | --- | --- |
| Title and abstract | 1 | (*a*) Indicate the study’s design with a commonly used term in the title or the abstract | 1 |
|  |  | (*b*) Provide in the abstract an informative and balanced summary of what was done and what was found | 1 |
| Introduction | | |  |
| Background/ rationale | 2 | Explain the scientific background and rationale for the investigation being reported | 3 |
| Objectives | 3 | State specific objectives, including any prespecified hypotheses | 3 |
| Methods | | |  |
| Study design | 4 | Present key elements of study design early in the paper | 3-4 |
| Setting | 5 | Describe the setting, locations, and relevant dates, including periods of recruitment, exposure, follow-up, and data collection | 3-4 |
| Participants | 6 | (*a*) Give the eligibility criteria, and the sources and methods of selection of participants | 4 |
| Variables | 7 | Clearly define all outcomes, exposures, predictors, potential confounders, and effect modifiers. Give diagnostic criteria, if applicable | N/A |
| Data sources/ measurement | 8* | For each variable of interest, give sources of data and details of methods of assessment (measurement). Describe comparability of assessment methods if there is more than one group | *4* |
| Bias | 9 | Describe any efforts to address potential sources of bias | 4 |
| Study size | 10 | Explain how the study size was arrived at | N/A |
| Quantitative variables | 11 | Explain how quantitative variables were handled in the analyses. If applicable, describe which groupings were chosen and why | N/A |
| Statistical methods | 12 | (*a*) Describe all statistical methods, including those used to control for confounding | N/A |
|  |  | (*b*) Describe any methods used to examine subgroups and interactions | N/A |
|  |  | (*c*) Explain how missing data were addressed | N/A |
|  |  | (*d*) If applicable, describe analytical methods taking account of sampling strategy | N/A |
|  |  | (*e*) Describe any sensitivity analyses | N/A |
| Results | | |  |
| Participants | 13* | (a) Report numbers of individuals at each stage of study—eg numbers potentially eligible, examined for eligibility, confirmed eligible, included in the study, completing follow-up, and analysed | 5 |
|  |  | (b) Give reasons for non-participation at each stage | N/A |
|  |  | (c) Consider use of a flow diagram | N/A |
| Descriptive data | 14* | (a) Give characteristics of study participants (eg demographic, clinical, social) and information on exposures and potential confounders | 5 |
|  |  | (b) Indicate number of participants with missing data for each variable of interest | N/A |
| Outcome data | 15* | Report numbers of outcome events or summary measures | 5-8 |
| Main results | 16 | (*a*) Give unadjusted estimates and, if applicable, confounder-adjusted estimates and their precision (eg, 95% confidence interval). Make clear which confounders were adjusted for and why they were included | N/A |
|  |  | (*b*) Report category boundaries when continuous variables were categorized | N/A |
|  |  | (*c*) If relevant, consider translating estimates of relative risk into absolute risk for a meaningful time period | N/A |
| Other analyses | 17 | Report other analyses done—eg analyses of subgroups and interactions, and sensitivity analyses | 5-8 |
| Discussion | | |  |
| Key results | 18 | Summarise key results with reference to study objectives | 8 |
| Limitations | 19 | Discuss limitations of the study, taking into account sources of potential bias or imprecision. Discuss both direction and magnitude of any potential bias | 10-11 |
| Interpretation | 20 | Give a cautious overall interpretation of results considering objectives, limitations, multiplicity of analyses, results from similar studies, and other relevant evidence | 8-11 |
| Generalisability | 21 | Discuss the generalisability (external validity) of the study results | 10-11 |
| Other information | | |  |
| Funding | 22 | Give the source of funding and the role of the funders for the present study and, if applicable, for the original study on which the present article is based | 1 |
